# Polygonal non-wetting droplets on microtextured surfaces

**DOI:** 10.1038/s41467-022-30399-0

**Published:** 2022-05-13

**Authors:** Jing Lou, Songlin Shi, Chen Ma, Xiaohuan Zhou, Dong Huang, Quanshui Zheng, Cunjing Lv

**Affiliations:** 1 Department of Engineering Mechanics and Center for Nano and Micro Mechanics, AML, Tsinghua University, Beijing, 100084 China; 2 National Key Laboratory of Science and Technology on Micro/Nano Fabrication, Institute of Microelectronics, Peking University, Beijing, 100871 China

**Keywords:** Fluids, Wetting, Surface patterning

## Abstract

Understanding the interactions between liquids and solids is important for many areas of science and technology. Microtextured surfaces have been extensively studied in microfluidics, DNA technologies, and micro-manufacturing. For these applications, the ability to precisely control the shape, size and location of the liquid via textured surfaces is of particular importance for the design of fluidic-based systems. However, this has been passively realized in the wetting state thanks to the pinning of the contact line, leaving the non-wetting counterpart challenging due to the low liquid affinity. In this work, confinement is imposed on droplets located on well-designed shapes and arrangements of microtextured surfaces. An active way to shape non-wetting water and liquid metal droplets into various polygons ranging from triangles, squares, rectangles, to hexagons is developed. The results suggest that energy barriers in different directions account for the movement of the contact lines and the formation of polygonal shapes. By characterizing the curvature of the liquid-vapour meniscus, the morphology of the droplet is correlated to its volume, thickness, and contact angle. The developed liquid-based patterning strategy under active regulation with low adhesion looks promising for low-cost micromanufacturing technology, DNA microarrays, and digital lab-on-a-chip.

## Abstract (web summary)

Exploring the interactions between liquids and solids is critical for improving control over fluidic systems. Here, authors develop an active way to tailor various polygonal shapes of non-wetting droplet on microtextured surfaces, resulting from the anisotropic energy barriers of the contact line.

## Introduction

The precise shaping of liquids based on thin films and droplets has attracted great interest due to its fundamental importance in understanding capillarity, as well as for practical applications in industry. Surface chemistry and roughness are widely employed for controlling the morphology of droplets to yield two typical wetting states: Wenzel^1^ and Cassie-Baxter^2^ wetting states. Droplets in the Cassie-Baxter wetting state are obtained by enhancing the roughness of surfaces characterized by hydrophobicity. The Cassie-Baxter state is significantly advantageous for self-cleaning^3–7^, anti-icing^8–10^, drag reduction^11^, heat transfer^12^, water harvesting^13^, and condensation^14–16^ since liquids have high repellency with surfaces. Liquids in the Wenzel wetting state present a completely wetting solid-liquid contact area, desirable features for pesticide retention on plant surfaces^17^ and boiling heat transfer^18^. Using highly enhanced capillarity and contact line pinning, spectacular properties of the Wenzel wetting state have been discovered, including directional and multilayer liquid spreading, as well as the formation of polygonal thin-liquid films and droplets^19–28^. For instance, Courbin et al.^20,21^ investigated deposited liquids on microtextured surfaces satisfying the imbibition condition and showed that they can form square, hexagonal, octagonal, and circular-shaped thin-liquid films. Raj et al.^25^ showed the ability to tailor various droplet contact area shapes ranging from squares, rectangles, hexagons, octagons to dodecagons by designing microtextures or through chemical heterogeneity on surfaces. Moreover, polygonal liquid films were also observed during the wetting transition from the Cassie-Baxter to the Wenzel state on textured surfaces^29–32^.

Nevertheless, the ability to shape liquid films and droplets into polygonal morphologies is mainly limited to wetting cases via passive ways, and creating polygonal droplets on non-wetting surfaces remains largely unexplored. The reason for this has to do with the difficulty of precisely shaping non-wetting liquids into desired patterns when compared to the wetting counterpart. The challenges lie in several aspects. First, liquid fronts can proceed spontaneously for complete and partial wetting cases^19–28^. However, thermodynamics suggests that capillary imbibition cannot take place on non-wetting surfaces^33^. Second, the contact line pinning for the non-wetting state (i.e., Cassie-Baxter state) is weak. Even though faceted droplets have been obtained by drop impingement on textured superhydrophobic surfaces ^34,35^, the appearance of droplet shape is transient and cannot be maintained for a long time. Third, the Cassie-Baxter wetting state is typically metastable and fragile, thereby can easily transfer to the Wenzel wetting state under perturbations such as vibration, pressure, drop impingement and evaporation^7,29–32,36–39^, making it difficult to realize morphology evolvement of droplets while maintaining the nonwetting state. Therefore, developing active ways to shape non-wetting droplets is still very challenging, but remains essential for a scientific breakthrough. Clarifying how to shape polygonal droplets in the non-wetting state may bring novel features and great prospects for engineered fluidic-based systems.

In this work, by contrast to polygonal liquid morphogenesis passively generated by varying the shape of the micropillars and the arrangement of the arrays in the wetting case, an active way to precisely shape non-wetting water and liquid metal droplets into various polygonal patterns by varying the thickness of the droplets is developed. High micropillars are employed and the minimum thickness of the droplets is typically more than two times of the pillar size, which guarantees a stable Cassie-Baxter wetting state. By imposing confinement on the droplets, the movement of the solid-liquid-vapour three-phase contact line in preferential directions is observed, shaping the droplets into triangles, squares, rectangles, and hexagons. The number of sides of the final polygonal pattern is found mainly dependent of the arrangement of the microtextures, while the shape of the microtexture cross-section plays a secondary role. The formation of the polygonal droplets is interpreted from an energy point of view: the three-phase contact lines preferentially moved in certain directions to more easily overcome the energy barrier due to the arrangement of the microtextures. A quantitative relationship correlating the polygonal pattern to the contact angle, the thickness of the droplet, and the geometric topology of the microtextures are established.

## Results

### Design of silicon micropillar arrays and operations

**Fig. 1:**
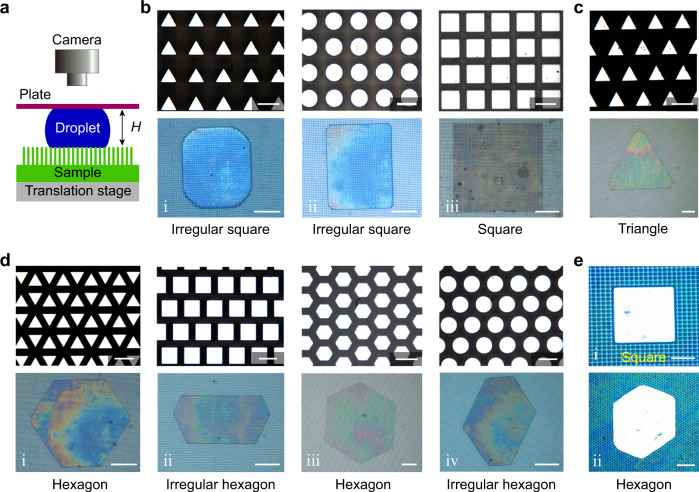
Images of the experimental setup, various cross-sectional areas of micropillars, arrangements of arrays, and shapes of liquid film patterns. **a** Schematic representation of the experimental setup used to perform confinement on the droplet (not drawn to scale). **b** Square and irregular square water droplet patterns created by the square arrangement of microtextures with triangular, circular, and square cross-sections (Supplementary Movie 1–3). **c** Triangular water droplet pattern created by a triangular arrangement of microtextures with a triangular cross-section (Supplementary Movie 4). **d** Hexagonal and irregular hexagonal water droplet patterns created by the hexagonal arrangement of microtextures with triangular, square, hexagonal, and circular cross-sections (Supplementary Movie 5–8). **e** Square and hexagonal liquid metal (Galinstan) droplet patterns, where (**e**–**i**) and (**e**–**ii**) have the same geometrical topologies (cross-section of micropillar and arrangement of array) as (**b**–**ii**) and (**d**–**i**), respectively. Scale bars for the arrays, 20 μm. Scale bars for the droplet patterns, 500 μm for (**b**–**d**) and 200 µm for (**e**).

A schematic representation of the experimental setup is given in Fig. 1a. It consisted of a *z*-axis translation stage on the bottom and a superhydrophobic glass plate (treated with a commercial coating agent, Glaco Mirror Coat “Zero”, Soft 99, Co.)^7,40^ on the top (Supplementary Method 1, Supplementary Fig. 1). As shown in the first row of each panel (Fig. 1b–d), scanning electron microscopy (SEM) illustrated well-defined shapes of silicon micropillar arrays fabricated by standard contact photolithography and deep-reactive ion etching^41^. These microtextured surfaces can be categorized into three groups. First, cross-sectional areas of the pillars based on triangular, circular, and square were arranged in square arrays, respectively (Fig. 1b). Second, the triangular cross-sections of the pillars were arranged in triangular arrays (Fig. 1c). Third, cross-sections of the pillars based on triangular, square, hexagonal, and circular were arranged in hexagonal arrays, respectively (Fig. 1d). Note that *a* refers to the typical length scale of the cross-sectional area of a pillar, i.e., the side length of the triangular and square pillars, the diameter of the cylindrical pillars, and the diagonal length of the hexagonal pillars (Supplementary Fig. 2). Also, the side length of the triangular and square pillars was set to *a* = 20 μm, side length of the hexagonal pillars was *a* = 10 μm, and diameter of the cylindrical pillars was *a* = 20 μm. The minimum spacing of the pillars was denoted as *b* = 10 μm and the pillar height was *h* = 90 μm (Supplementary Method 2, Supplementary Table 1). All samples were first coated by a silane film CF_3_(CF_2_)_9_CH_2_CH_2_SiCl_3_ (FDDTS) deposited by RPX-540 of Integrated Surface Technologies, Inc. Afterward, the surface of each pillar became hydrophobic with the Young contact angle *θ*_0_ = 110 ± 5°. Each sample was cleaned before the experiments. By placing water (Fig. 1b–d) and liquid metal (Galinstan, Fig. 1e) droplets in a confinement consisting of a microtextured surface on the bottom and a superhydrophobic glass surface on the top, polygonal-shaped droplets were formed by narrowing the separation of the two surfaces. The top-view visualization of the droplet by a CCD camera (Canon 550D, Hi-Scope advanced KH3000) and separation *H* of the two surfaces by length sensor (Heidenhain-Metro 2581) were recorded simultaneously. The details of the microfabrication, silanization, sample-cleaning procedure, contact angle measurements, and experimental process are all provided in the Methods section.

### Polygonal shapes of droplets and energy barrier

As shown in Fig. 1b–e, various polygonal shapes of droplets were obtained by varying the shape of the cross-sectional area of the pillars and the arrangement of the arrays. The second row in Fig. 1b–d shows the corresponding droplet patterns. Several features can be extracted. First, the droplet revealed a pattern mainly determined by the arrangement of the arrays, while the cross-sectional area of the microtexture is likely to be of secondary importance. In particular, square droplet patterns were created for microtextures arranged in square arrays regardless of the shape of the pillar cross-sectional area (Fig. 1b). Similar cases were also applicable for Fig. 1c–e. As shown in Fig. 1e, square and hexagonal liquid metal droplets formed on the surfaces shown in Fig. 1b(ii) and Fig. 1d(i), respectively (Supplementary Discussions 1 and 2). Moreover, irregular polygonal patterns can be obtained because of anisotropic energy barriers creating preferred spreading directions as discussed further in the text. Second, a comparison of the cross-sectional areas of the pillars and the arrangements of the arrays revealed that the shapes of the droplet patterns demonstrated in Fig. 1b–e were consistent with those of the liquid films created on wetting microtextured surfaces^20,25^. Third, remarkably different from the wetting case^19–28^, when the confinement is removed, the polygonal droplet reverts back to its initial spherical shape (Supplementary Discussion 3, Supplementary Fig. 5), which shows a good reversibility of the wetting state transition. Moreover, there is a robust reproducibility of the polygonal droplet pattern when the confinement is repeatedly imposed and removed (Supplementary Discussion 4, Supplementary Figs. 6 and 7).

By compressing the droplet to decrease its thickness *H* (Supplementary Movie 1), the boundary of the droplet expanded more easily along several specific directions, leading to the formation of corners and straight sides until polygonal droplet patterns arose. Note that the solid-liquid contact area was set in a stable Cassie-Baxter wetting state during the whole experiment^40^, and the solid-liquid-vapour three-phase contact line was located on the top of the pillars. In the following, more focus was paid to answering the key question on how non-wetting polygonal droplet patterns were formed.

**Fig. 2:**
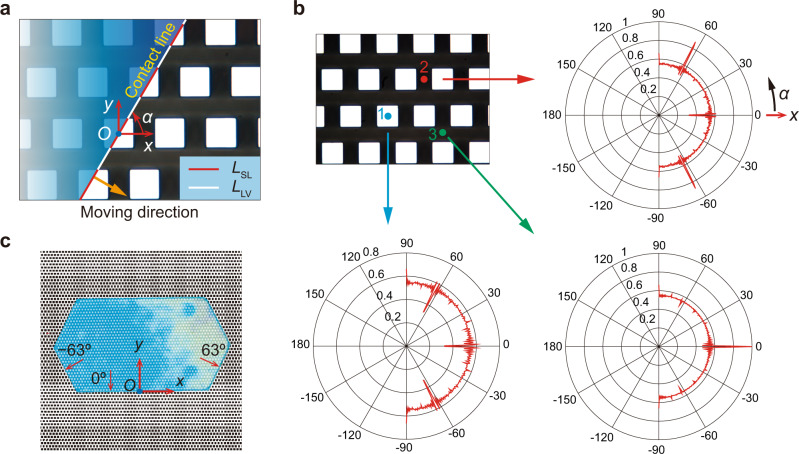
Moving direction of the solid-liquid-vapour three-phase contact line. **a** Schematic representation of the pillars (white color) and contact line from a top view. The dot marked with “*O*” represents the reference point. The moving direction of the three-phase contact line is marked using an orange arrow. The red and white lines represent the solid-liquid contact line *L*_SL_ and liquid-vapour part *L*_LV_, respectively. *α* is defined as the angle (having a positive value in anticlockwise direction) between the contact line and the *x*-axis. **b** Diagram illustrating the liquid-vapour line fraction *ε*(*α*) = *L*_LV_/*L* as the function of *α* in the polar coordinate system for different reference points. **c** Final configuration of the droplet (Supplementary Movie 9). The block directions (red arrows) *α* ≈ 63°, 0°, and – 63° are obtained from (**b**). Source data are provided as a Source Data file.

As shown in Fig. 1, the side lengths of the polygonal droplet patterns were approximated as infinitely long straight lines since they were much larger than the size of a single micropillar. Hence, the three-phase contact line length can be expressed as: *L* = *L*_SL_ + *L*_LV_ (see Fig. 2a) on each side consisting of two parts. The first part (red color) *L*_SL_ was in contact with the top of the pillars, and the second part (white color) *L*_LV_ was pending in air. The liquid-vapour line fraction was defined as: *ε* = *L*_LV_/*L* ( ≤ 1). Thus, the moving behavior of the contact line and underlying physics can be understood along the following line of thought. As exemplified in Fig. 2a, the center of one pillar was considered as a reference point (marked with “*O*”), and the angle between the positive direction of the *x*-axis and the direction along the contact line was defined as *α*. The positive value of *α* (anticlockwise direction) was marked in Fig. 2a. Basically, the contact line could move in an infinite number of directions (e.g., the yellow arrow denotes one of these directions), but *ε*(*α*) may be different along these directions. Therefore, the contact line would move according to the path with the lowest energy barrier. Specifically, small values of *ε*(*α*) (or larger solid-liquid line fraction 1 – *ε*(*α*) = *L*_SL_/*L*) would decrease the surface energy (or larger ∆*E*(*α*) = –*L*[1 – *ε*(*α*)] *γ*_LV_cos*θ*_0_d*x*), so that the contact line can move easily. Here, *γ*_LV_ and d*x* were defined as the liquid-vapour interfacial tension and element of the displacement along the moving direction, respectively. By contrast, larger *ε*(*α*) would block the contact line in the direction of *α*. Next, *ε*(*α*) can easily be calculated according to the morphology of the pillars to explain the formation of the polygonal droplets. However, ∆*E*(*α*) also depended on the location of the contact line (or the reference point). Note that an infinite number of reference points exist, for the sake of simplicity, only three representative reference points (marked as 1, 2, and 3 in Fig. 2b) were used, and *ε*_1_(*α*), *ε*_2_(*α*), and *ε*_3_(*α*) will be calculated.

As shown in Fig. 2b plotted in polar coordinates, three diagrams *ε*_1_(*α*), *ε*_2_(*α*), and *ε*_3_(*α*) were drawn as a function of *α*. First, the relationship between *ε*_1_(*α*) and *α* for reference point 1 was checked. As expected, some maximum values of *ε*_1_(*α*) (*ε*_1max_(*α*) ≈ 0.66 at *α* ≈ 90°, 0°, and –90°) were obtained. For reference point 2, obviously *ε*_2max_(*α*) ≈ 0.87 at *α* ≈ 63° and –63°. For reference point 3, *ε*_3max_(*α*) = 1.0 appeared at *α* = 0°. The combination of reference points 1, 2, and 3 suggested that *α* ≈ 0°, 63°, and –63° corresponded to the three highest values of *ε*, and the most possible directions to block the three-phase contact lines. As shown in Fig. 2c, a total of six directions along which the side of the droplet would mostly be blocked were obtained since the *y*-axis was set as the axis of surface symmetry. This finally resulted in a hexagonal droplet pattern shape. In sum, the above explanations suggested consistency between the theoretical analyses and experimental results. Thus, the formation of a polygonal droplet pattern resulted from the energy barrier in certain movement directions of the liquid. Basically, an infinite number of reference points existed, but the underlying physics would not change. The above-illustrated method can be used as a universal way to predict the droplet patterns for other cases, such as the outcomes in Fig. 1b–e (Supplementary Discussion 5, Supplementary Fig. 8).

### Estimation of advancing contact angle and meniscus curvature

**Fig. 3:**
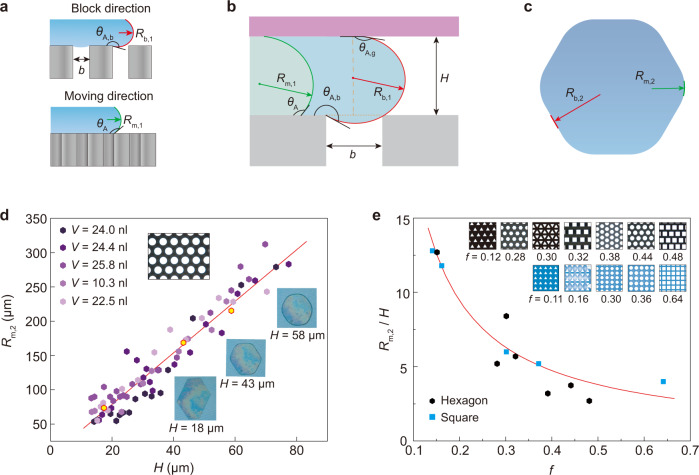
Liquid profile, geometrical parameters and evolution of the liquid film. **a** Schematic illustration of the block (red arrow) and moving (green arrow) directions of the liquid profile from the side view. *R*_b,1_ and *R*_m,1_ denote the curvature radii of the red and green curves, respectively. **b** Close-up showing the liquid profile with relevant geometrical parameters. *b* denotes the spacing of the pillar, and *H* the separation between the upper glass plate and the pillar. *θ*_A_ and *θ*_A,b_ denote the advancing contact angles along the moving and block directions, respectively. *θ*_A,g_ denotes the advancing contact of the glass plate. **c** Schematic illustration of the principal curvature radii *R*_b,2_ and *R*_m,2_ of the liquid-vapour menisci from the top view. **d** Evolution of the hexagonal droplet pattern with *H* and *R*_m,2_. The dots are experimental data with different volumes, and the red line is obtained using the best fit (*R*_m,2_/*H* ≈ 3.7). Insets showing the appearances of the surface and the representative liquid patterns (corresponding to the yellow dots). **e** The variations of *R*_m,2_/*H* as a function of *f* for various cross-sections of the pillars and arrangements of the arrays (see insets), as well as the droplet shape. The dots are experimental data and the red curve represents the theoretical results obtained by Eq. (3). Source data are provided as a Source Data file.

On non-wetting textured surfaces, the apparent contact angle *θ* of a droplet can be quantified by employing the Cassie-Baxter equation^2^

1 $$\cos \theta =-1+f(1+\,\cos {\theta }_{0})$$

where *f* is the solid-liquid area fraction (Supplementary Table 1). By considering the formation of droplet pattern for contact line moving forward, *θ*_0_ and *θ* were respectively replaced by *θ*_0,A_ and *θ*_A_ in Eq. (1), with *θ*_0,A_ and *θ*_A_ representing the advancing contact angles on flat and textured surfaces^25,36^, respectively. In our experiment, the advancing contact angle *θ*_A_ of FDDTS treated smooth silicon surface was defined as *θ*_0,A_ = 120 ± 5°. A sketch showing the wetting states around the contact lines is depicted in Fig. 3a. Afterward, “block direction” was used to denote the direction of *α* for *ε*(*α*) with local maximum value (*ε*_max_(*α*), see Fig. 2). By comparison, the other directions were called “moving direction”. However, the contact line in the block direction could also move when the droplet was compressed enough and the energy barrier ∆*E*(*α*) was overcome. Along the moving direction (Fig. 3b), the advancing angle *θ*_A_ remained nearly constant and quantified by Eq. (1). Along the block direction (Fig. 3b), the contact line pined on edge of the pillars, and the liquid-vapour meniscus bulged forward before touching down onto the next row of pillars^42^. Consequently, Eq. (1) cannot predict the advancing contact angle *θ*_A,b_ (*θ*_A,b_ > π > *θ*_A_).

The side-views of the droplet profile in the moving and block directions are illustrated in Fig. 3b. For the liquid-vapour meniscus moving forward to touch the next row of a pillar in the block direction, the advancing contact angle *θ*_A,b_ around the contact line at the pillar edge required reaching a maximum value (red curve)^42^. Since the spacing *b* was given and both the advancing contact angle of the upper superhydrophobic glass plate *θ*_A,g_ and separation *H* were measured experimentally, two unknown parameters *θ*_A,b_ and *R*_b,1_ can be obtained based on two geometrical relationships: *R*_b,1_ [cos(π – *θ*_A,b_) + cos(π – *θ*_A,g_)] = *H* and sin(*θ*_A,b_ – π) = *b*/(2*R*_b,1_). Here, *R*_b,1_ is one principal curvature radius of the meniscus. Since *θ*_A,g_ ≈ 170°, the approximation cos*θ*_A,g_ ≈ –1 can be used for simplification. Next, *R*_b,1_ = *H*/2 + *b*^2^/(8*H*) ≈ *H*/2 and sin *θ*_A,b_ = – 1/[*H*/*b* + *b*/(4*H*)] ≈ *b*/*H* can be obtained by considering *b* ≪ *H*.

By neglecting gravity, the Laplace pressure in the droplet would have a uniform value. Hence, the total curvature of the liquid-vapour meniscus can be kept constant everywhere, leading to

2 $$\frac{1}{{R}_{{{\mathrm{b}}},1}}+\frac{1}{{R}_{{{\mathrm{b}}},2}}=\frac{1}{{R}_{{{\mathrm{m}}},1}}+\frac{1}{{R}_{{{\mathrm{m}}},2}}$$

where *R*_b,2_ and *R*_m,2_ are the other two principal curvature radii of the menisci, respectively. As shown in Fig. 3c, the contact line of the block direction was close to a straight line, leading to *R*_b,2_ ≈∞  and 1/*R*_b,1_ ≈ 1/*R*_m,1_ + 1/*R*_m,2_. Moreover, the geometrical relationship in Fig. 3b yielded *R*_m,1_ [cos(π – *θ*_A_) + cos(π – *θ*_A,g_)] = *H*, suggesting *R*_m,1_ ≈ *H*/(1 – cos*θ*_A_). A substitution of *R*_m,1_, *R*_b,1_, and *R*_b,2_ into Eq. (2) would yield

3 $${R}_{{{\mathrm{m}}},2}\approx \frac{H}{1+\,\cos {\theta }_{{{\mathrm{A}}}}}$$

where *b* ≪ *H* was employed to satisfy most cases of our experiments. In fact, the minimum *H* in our experiments was estimated to be about 2*b* since a smaller value of *H* would trigger the breakdown of superhydrophobic state^40^.

Using Eq. (3), the corner curvature radius *R*_m,2_ on a given microtextured surface should be proportional to the separation *H* of the two surfaces for a specific value of *θ*_A_. In Fig. 3d, the results of Eq. (3) were confirmed by exhibiting a hexagonal droplet pattern created on a hexagonally arrayed surface consisting of cylindrical pillars. The geometrical parameters of the pillars were *a* = 17.4 μm, *b* = 7.6 μm (*f* = 0.44), and *h* = 90 μm. Droplets with volumes ranging from *V*_0_ = 10 nl to 30 nl were employed. The best fit yielded *R*_m,2_/*H* ≈ 3.7, confirming an independent relationship with the droplet volume. By substituting *f* = 0.44 and *θ*_0,A_ = 120° in Eqs. (1) and (3), *θ*_A_ ≈ 141° and *R*_m,2_/*H* = 1/(1 + cos*θ*_A_) ≈ 4.5 were obtained, close to the fitted experimental values. Furthermore, Eq. (3) was tested on a series of squarely and hexagonally arrayed micropillars with various geometries (area fraction *f* and cross-sections, such as circular, triangular, and square pillars) and volumes (*V*_0_ = 10 nl to 50 nl). In this case, *θ*_A_ is the function of *f* and can be determined via cos *θ*_A_ = – 1 + *f* (1 + *θ*_0,A_) since *θ*_0,A_ is known. Thus, we can obtain the relationship between *R*_2,m_/*H* and *f* through Eq. (3). As demonstrated in Fig. 3e, Eq. (3) finely followed the experimental data regardless of the cross-section of the pillar and the arrangement of the array, shape of the droplet pattern, or droplet volume.

**Fig. 4:**
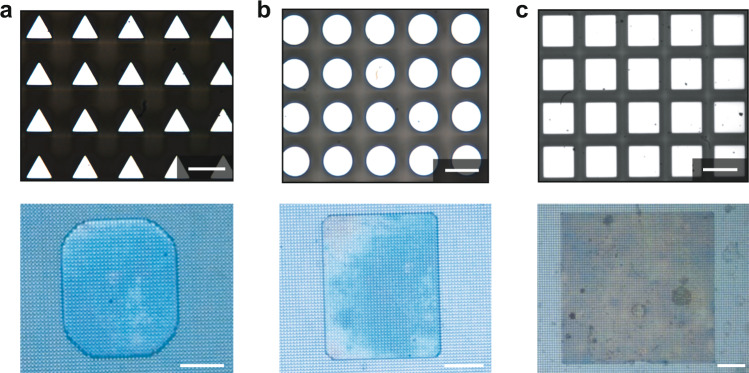
Resolution of the droplet patterns on micropillar surfaces with various pillar spacings. From (**a**–**c**), *b* = 11 μm, 8 μm, and 7 μm, respectively. By approaching *H*_c_, the corners of the droplet patterns gradually sharpened. Scale bar of the pillars, 20 μm. Scale bar of the droplet patterns, 500 μm.

The physics we developed in Eq. (3) was useful in many cases. By considering *θ*_A_ constant for a specific surface, the compression of the droplet would reduce *H*, as well as decrease *R*_m,2_. This meant the corner of the polygonal droplet became gradually sharper. In other words, the minimum value of *H* would determine how sharp the droplet pattern could attain. Our previous work^40^ showed a decline in *H* to a critical (maximum) value *H*_c_ (about 2*b*), the droplet suddenly penetrated the gap between pillars, and the polygonal droplet pattern disappeared. To design polygonal droplet patterns with sharper corners, the spacing *b* of the pillars should reduce; otherwise, passivated polygonal droplet patterns will be obtained. This conclusion was consistent with Fig. 4: when *H* approaches *H*_c_, the corners of the droplet patterns become gradually sharper on the pillared surfaces with spacings of *b* = 11 μm, *b* = 8 μm, and *b* = 7 μm.

### Modeling of droplet pattern

**Fig. 5:**
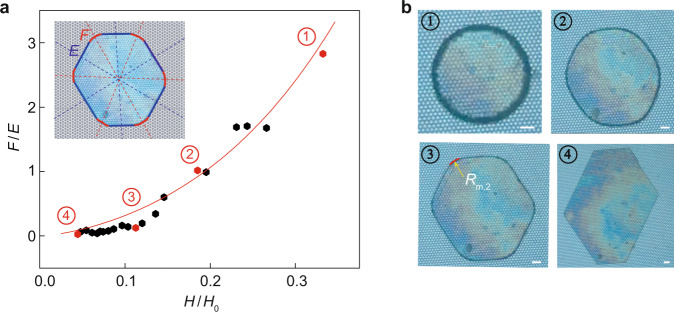
Evolution of the droplet pattern on the surface with cylindrical pillars. The pillars are arranged in hexagonal patterns. The diameter of the cross-sectional area, the spacing and the height of the cylindrical pillars are 17.4 μm, 7.6 μm and 90 μm, respectively. **a** Relationship between *F*/*E* and *H*/*H*_0_, where *H*_0_ = 318 μm and *V*_0_ = 23 nl. The inset defines the contact line lengths of the side (*E*, blue color) and corner (*F*, red color) of the droplet. The dots represent the experimental data, and the red curve is the theoretical results obtained by Eq. (4). **b** Selected images with four different values of *H*, corresponding to *H* = 106 μm, 59 μm, 36 μm, and 14 μm, respectively. The experimental data of the four images in (**b**) are marked in red in (**a**). Scale bar, 100 μm. Source data are provided as a Source Data file.

As shown in the inset of Fig. 5a, the compression of the droplet promoted the formation of a polygon by dissimilar *θ*_A_ and *θ*_A,b_, with sides and corners can clearly be identified (Supplementary Movie 8). Here, *E* and *F* were defined as the lengths of the contact line of the side (blue color) and corner (red color) of the droplet^25^, respectively. To quantify the droplet shape transition, the variation in corner-side length ratio *F*/*E* as a function of the relative height *H/H*_0_ was verified. In this case, *H*_0_ represents the initial value of *H* with a constant value. Here, *H*_0_ was selected as the height of the droplet before contact with the upper superhydrophobic glass plate.

Based on the projected contour of the droplet from the top view with a one-sixth circle on each corner, a relationship between *E* and *F* was established by employing the liquid volume conservation: *V*_0_/*H* ≈ (3^3/2^/2)*E*^2^ + 6*ER*_m,2_ + π*R*_m,2_^2^, where *F* = π*R*_m,2_/3. More detailed derivation is given in Supplementary Discussion 6 and Supplementary Fig. 9. Further simple calculations correlated *F*/*E* to the intrinsic parameters of the system, such as *V*_0_, *H*, and *θ*_A_

4 $$\frac{F}{E}\approx \frac{3}{\sqrt{1+\frac{3{V}_{0}}{{H}^{3}}{(1+\cos {\theta }_{{{\mathrm{A}}}})}^{2}}-1}$$

As shown in Fig. 5a (red curve), the results obtained by Eq. (4) agreed well with the experimental data. Equation (4) also led to two scaling regimes. First, very small thicknesses of the droplet (i.e., *H*/*H*_0_ ≪ 1) yielded *F*/*E* ~ (*H*/*H*_0_)^3/2^. Second, comparable *H* to *H*_0_ yielded *F*/*E* ~ (*H*/*H*_0_)^3^ (Supplementary Discussion 6). Similar analyses can be carried out for the liquid film pattern with other shapes.

## Discussion

**Fig. 6:**
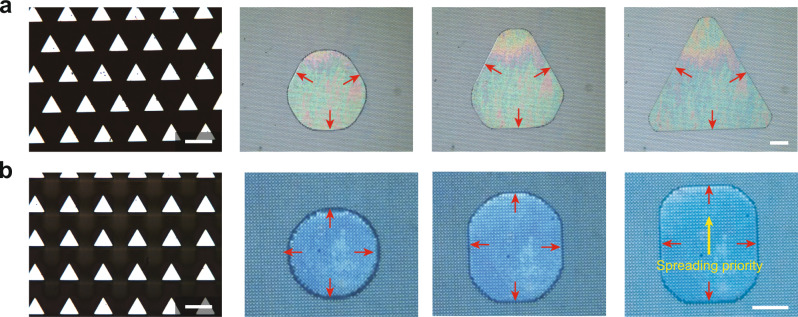
Evolution of the morphology and spreading priority of the contact line on surfaces with a triangular cross-section of the pillars and various arrangements of the arrays. The red arrows represent the block directions. **a** The liquid spreads synchronously (Supplementary Movie 4). **b** One side (top side) of the square liquid moves along the direction (yellow arrow) pointing at one vertex of a single pillar (Supplementary Movie 1). Scale bar of the pillars, 20 μm. Scale bar of the droplet patterns, 250 μm.

As shown in Fig. 6, the droplet pattern during compression formed in a specific way even though its final shape was determined by the arrangement of the pillars. Here, more focus was paid to the spreading priority of the contact line. On the surface consisting of triangular pillars arranged in triangular arrays (Fig. 6a), the triangular droplet pattern was formed and the three sides spread synchronously (Supplementary Movie 4). In Fig. 6b, the surface consisting of triangular pillars was arranged in square arrays (Fig. 6b). During the formation of the square droplet pattern (Fig. 1b), the four sides did not spread synchronously (Supplementary Movie 1). One side of the droplet pattern was parallel to one side of the pillars pins, but the opposite side moved pointing to one vertex of the pillars (yellow arrow). Thus, the final shape of the droplet pattern and its evolution were influenced by the cross-section of the pillar and the arrangement of the array, respectively (Supplementary Discussion 7, Supplementary Movie 10).

However, open questions remain. As shown in Fig. 1, it seems that the droplet seeks regular square (Fig. 1b(iii)), triangular (Fig. 1c) and hexagonal (Fig. 1(d)(iii)) shapes only for square, triangular and hexagonal pillars arranged on arrays having the same symmetry. For other cases, irregular liquid films form. For instance, as shown in Fig. 5b, pillars with the circular cross-sectional area are employed. Even the pillars are arranged in hexagonal arrays, when the droplet was more compressed, its shape deviates more from a regular hexagon. There are many factors that may account for the morphology evolution of the liquid film. The inevitable existence of contact angle hysteresis and contact line pinning may play a non-negligible role for the anisotropic spreading of the liquid film. Moreover, when the symmetry of the cross-sectional area of individual pillars is not consistent with the symmetry of the array of the pillars (e.g., Fig. 1b(i)(ii), Fig. 1d(i)(ii)(iv)), anisotropic energy barriers generate in different spreading directions of the contact line that, in turn, exacerbates the asymmetric evolution of the liquid film. In the experiment, concerning the transverse size (10^2^ – 10^3^ μm) of the compressed liquid film is more than one order of magnitude compared to its thickness (~ 10 μm), it is very challenging to make the upper glass plate and lower pillared surface perfectly parallel to each other. In addition, the upper superhydrophobic glass plate may not be sufficiently flat after being coated by a layer of the commercial coating agent. Detailed study of these influences and the evolution of the droplet would deserve a dedicated study, and more work would be required to clarify the reason accounting for the asymmetry of the droplet pattern. Lastly, similarity and difference between water and liquid metal, as well as the above influences, remain to be further understood and explored.

In sum, an interesting and hitherto unreported strategy was developed to actively shape droplets on nonwetting microtextured surfaces. This work breaks through our understandings that polygonal liquid patterns could only be attainable on wetting textured surfaces with the help of highly enhanced capillarity and contact line pinning. This controllable and highly repeatable route for forming versatile droplet patterns via confinement, as well as detailed fundamental findings of the physics would provide a new method for liquid-based manufacturing technologies. As an example, after the liquid metal is generated, a decrease of its temperature is able to make solidification of the polygonal pattern (Supplementary Fig. 3), which is promising to be extended to other liquid-microtextured systems for specific applications such as forming a small-scale pattern with predefined shape. It is believed that the strategy reported in this paper can find unique applications in lithography, miniaturized biochips, and smart windows, among others.

## Methods

### Sample fabrication and coating

Standard contact photolithography and deep reactive ion etching were first used to create silicon micropillar arrays. A single layer molecule CF_3_(CF_2_)_9_CH_2_CH_2_SiCl_3_ (FDDTS) was then deposited on the micropillar-patterned surface by CVD (RPX-540 of Integrated Surface Technologies, Inc.). The Young contact angle of the FDDTS coating was *θ*_0_ = 110 ± 5°. Before the start of the experiment, all samples were rinsed with acetone and alcohol (1 min, respectively) followed by deionized water and blow drying with pure nitrogen.

### Small droplet preparation

A sterile hypodermic needle (23 G, 0.6 × 25 mm) was used to generate water droplets. To create small droplets, the needle was first treated with a commercial coating agent (Glaco Mirror Coat “Zero”, Soft 99, Co.) five times to make the wall of the needle superhydrophobic. After that, water droplets with the volume of nanoliter were able to be generated from the needle.

### Characterization of surfaces

The configuration of the micropillar-patterned surfaces was characterized by field-emission scanning electron microscopy (SEM) (Quanta 250 FEG). Details of the corresponding dimensions of samples are shown in Supplementary Table 1. The optical images were recorded by a CCD camera (Canon 550D).

### Contact angle measurements

The contact angle and contact angle hysteresis were measured at room temperature (about 25 °C) and relative humidity of 40%. An automatic microscopy contact angle meter (DataPhysics OCA25) was employed to measure the contact angle of small droplets (5 μl) deposited on a smooth silicon sample to yield *θ*_0_ = 110 ± 5°. By injecting and pumping backwater from the droplet, advancing *θ*_0,A_ = 120 ± 5° and receding contact angles *θ*_0,R_ = 100 ± 5° were obtained.

### Determination of separation *H*

The thickness of the droplet *H* was determined by first defining the value of the length sensor as *H*_0_ at the moment when the glass plate touched the top of the droplet, and a picture from the top was taken. Since the droplet was non-wetting (*θ* > 90°), the curvature radius *R* of the liquid-vapour meniscus of the droplet can directly be measured. Afterward, the droplet was compressed, and the length sensor simultaneously recorded the relative position of the glass plate (Supplementary Fig. 1). Finally, the value of the length sensor at the critical moment just before the collapse of the Cassie wetting state was defined as *H*_c_. Meanwhile, another picture was also taken from the top to obtain the sectional area *A* of the droplet. Since *R*, *A*, and the relative position Δ*H* = *H*_0_ – *H*_c_ were all known, the three unknown parameters *H*_0_, *H*_c_, and the droplet volume *V*_0_ can be obtained

5 $$\left\{\begin{array}{c}{V}_{0}=\frac{\pi }{3}{H}_{0}^{2}(3R-{H}_{0})=A{H}_{{{\mathrm{c}}}}\\ {H}_{0}=\varDelta H+{H}_{{{\mathrm{c}}}}\hfill\end{array}\right.$$

Since the length sensor recorded the relative height during the whole experimental process, the instantaneous thickness *H* of the droplet can be calculated after the determination of *H*_0_.

### Determination of curvature radius *R*_m,2_

Droplet images were first recorded by a CCD camera (Canon 550D) from the top view, and the contour of the droplet was then extracted. For hexagonal droplets (Fig. 3c), a quadratic curve was employed to fit the droplet contour at one corner of the droplet to yield curvature radius *R*_m,2_. Using the same process, the curvature radii of the other five corners were obtained, and the average value of these six curvature radii was calculated. For droplet patterns with other shapes, similar calculation steps were performed.

### Determination of lengths *E* and *F*

Based on the top view image, the contour of the droplet pattern was first extracted. The side length *E* and corner length *F* were then measured. Considering polygonal droplet patterns, the average values of *E* and *F* were used.

### Statistics & reproducibility

No statistical method was used to predetermine sample size. No data were excluded from the analyses. The experiments were not randomized. The Investigators were not blinded to allocation during experiments and outcome assessment.

## Supplementary information

Supplementary Information

Description of Additional Supplementary Files

Supplementary Movie 1

Supplementary Movie 2

Supplementary Movie 3

Supplementary Movie 4

Supplementary Movie 5

Supplementary Movie 6

Supplementary Movie 7

Supplementary Movie 8

Supplementary Movie 9

Supplementary Movie 10

## Data Availability

The data that support the findings of this study are available from the corresponding authors upon request. Source data are provided with this paper.

## Source data

Source Data
