# Supplementary material for: Polygonal non-wetting droplets on microtextured surfaces
Source: Nat Commun. 2022 May 13;13:2685. doi: 10.1038/s41467-022-30399-0 (PMC9106735; doi:10.1038/s41467-022-30399-0)
Supplement: Supplementary file 1 — Supplementary Information [file 41467_2022_30399_MOESM1_ESM.pdf]

Supplementary Information for

Polygonal non-wetting droplets on microtextured surfaces

Jing Lou[1], Songlin Shi[1], Chen Ma[1], Xiaohuan Zhou[1], Dong Huang[2], Quanshui Zheng[1], Cunjing Lv[1*]

[1]Department of Engineering Mechanics and Center for Nano and Micro Mechanics, Tsinghua University, Beijing 100084, China

[2]National Key Laboratory of Science and Technology on Micro/Nano Fabrication, Institute of Microelectronics, Peking University, Beijing 100871, China

Content

Supplementary Method 1. Experimental procedure

Supplementary Method 2. Sample details

Supplementary Discussion 1. Solidification of the liquid metal film

Supplementary Discussion 2. Result of mercury droplet

Supplementary Discussion 3. Reversibility of the wetting state transition due to confinement

Supplementary Discussion 4. Reproducibility of the polygonal droplet pattern

Supplementary Discussion 5. Diagram for more types of micropillared structures

Supplementary Discussion 6. Theoretical analysis of the geometry of the droplet

Supplementary Discussion 7. Spreading priority of the liquid

Supplementary Movie. Description of the Supplementary movie

[*] To whom correspondence should be addressed. Email: cunjinglv@tsinghua.edu.cn.

Supplementary Method 1. Experimental procedure

The experimental setup is shown in Supplementary Fig. 1a. Before the experiment was carried out, the setup was optimized to guarantee that the microtextured surface and the upper glass plate are parallel to each other. To realize this aim, the following procedures were carried out. First, the platform with the sample was adjusted by using an air level. Then, five droplets were deposited at five different positions of the sample (i.e., the center, and another four locations which are close to the corners of the sample), and smoothly decline the upper plate to compress these droplets. Meanwhile, the base plate was controlled by a high precision motorized goniometer (x-Zolix PSAG15-250, y-Zolix PSAG15-370). The spreading behaviors of these five droplets were monitored. When all of them were able to achieve same spreading behaviors, the upper plate and the bottom sample were believed to be sufficiently parallel to each other. After that, experiments and data recording data were carried out.

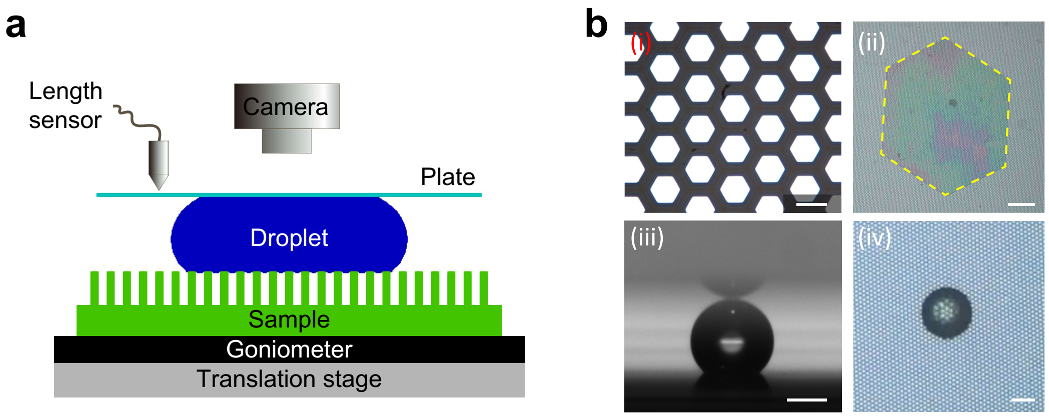

Supplementary Figure 1. Sketch of the experiment setup and the live experiment. (**a**) Schematic of the experimental setup (not drawn to scale) used to study polygonal droplet pattern formation. (**b**) Images showing details of the experiment: appearance of the sample (i); the final shape of the droplet pattern (ii); the side view (iii) and the top view (iv) images at the moment when the glass plate is close to the top of the droplet. Scale bars in (i)-(iv) are 20 μm, 400 μm, 200 μm and 200 μm, respectively.

Similar experimental procedures were carried out for liquid metal (Galinstan) droplets. To prevent oxidation of the liquid metal droplets, a big drop (H_2SO_4/water mixture [10% (v/v)]) was first deposited on the micropillared surfaces. Then, the superhydrophobic glass plate was used to press the big drop to make it in the Wenzel wetting state. After that, the upper glass plate was lifted up, and then a liquid metal droplet was deposited in the big drop and meanwhile on

the top of the pillars, where the volume of the liquid metal droplet was comparable to the experiments of water droplets as shown in Supplementary Fig. 1b. Since the intrinsic contact angle of the liquid metal droplet on the silicon surface in the H_2SO_4/water mixture environment was quite high (i.e., $\theta_0 = 154.3 \pm 1.6°$), the liquid metal droplet was in the Cassie-Baxter wetting state. After these steps were finished, similar experimental procedures (for water droplets) were carried out for liquid metal droplets.

Supplementary Method 2. Sample details

As shown in Supplementary Fig. 2, the top view of the microtextured surface employed in the experiments is shown. Specifically, Supplementary Fig. 2a demonstrates the triangular pillars arranged in hexagonal (circle 1), triangular (circle 2) and square (circles 3 and 4) arrays. The side length *a* of the pillar and the spacing *b* between the pillars are defined. As shown in Supplementary Fig. 2b-d, the appearances of the square, hexagonal and circular pillars arranged in various arrays are shown, respectively. In Supplementary Fig. 2c, *a* is defined as the distance between the two points on the diagonal of the hexagonal pillars. In Supplementary Fig. 2d, *a* is defined as the diameter of the circular pillars.

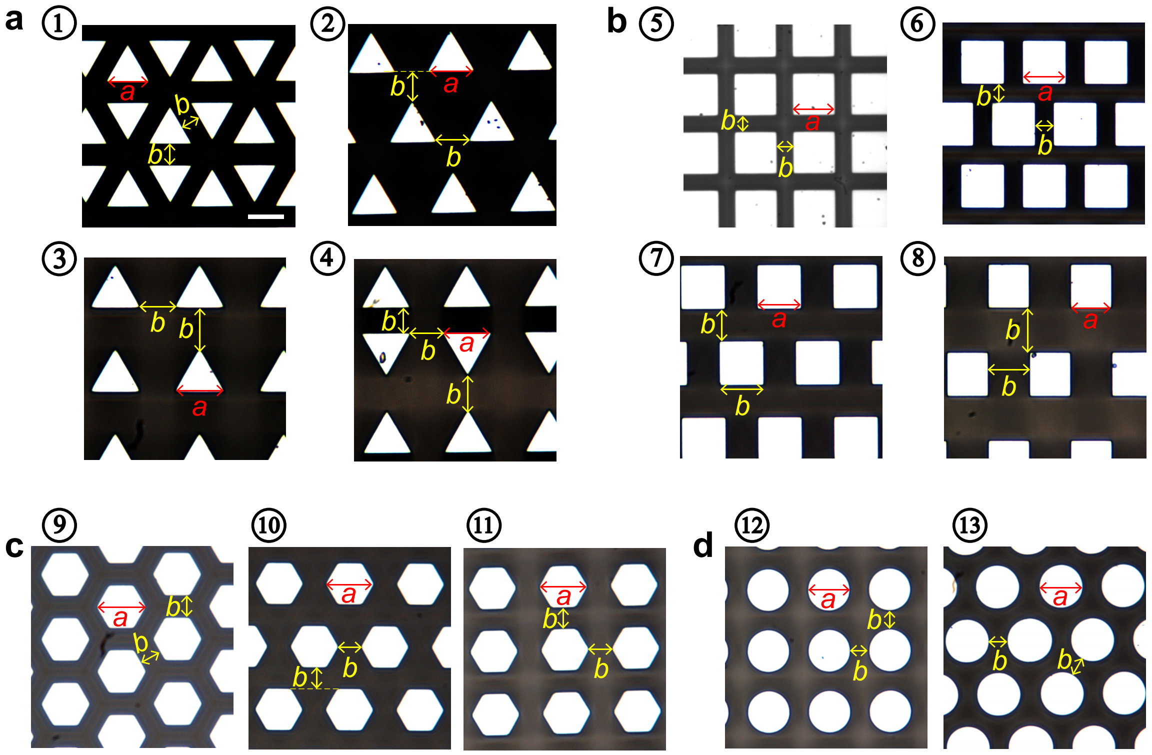

Supplementary Figure 2. Optical microscope images of the samples. Samples with triangular pillars (**a**), square pillars (**b**), hexagonal pillars (**c**) and circler pillars (**d**) arranged in various arrays. Scale bar in (a) is 20 μm, which is available for all the images.

In the following Supplementary Table 1, the geometrical parameters of the microtextures are measured and listed, and the definition of the solid-liquid area fraction *f* is given.

Supplementary Table 1. Geometrical parameters of the samples numbered in Supplementary Fig. 2.

No.	a (μm)	b (μm)	f	f (definition)
1	14.6	7.6	0.28	$f=\left[a/\left(a+\sqrt{3}b\right)\right]^2$
2	14.1	10.9	0.15	$f=\frac{\sqrt{3}}{4}a^2\bigg/\left[(a+b)(\frac{\sqrt{3}}{2}a+b)\right]$
3	14	11	0.14	
4	14.3	10.7	0.16	
5	18.7	6.3	0.56	$f=\left[a/(a+b)\right]^2$
6	17.4	7.6	0.48	
7	17.1	12.9	0.32	
8	17	18	0.24	
9	15	7.8	0.39	$f=\left[a\bigg/\left(a+\frac{2}{\sqrt{3}}b\right)\right]^2$
10	16.5	8.5	0.30	$f=\frac{3\sqrt{3}}{8}a^2\bigg/\left[(a+b)\left(\frac{\sqrt{3}}{2}a+b\right)\right]$
11	16.5	8.5	0.30	
12a	17.1	7.9	0.37	$f=\frac{\pi}{4}a^2\bigg/(a+b)^2$
12b	48.6	51.4	0.19	
13	17.4	7.6	0.44	$f=\frac{\pi}{2\sqrt{3}}a^2\bigg/(a+b)^2$

Supplementary Discussion 1. Solidification of the liquid film

Despite the reversibility and reproducibility of the wetting state, we are wondering whether the liquid pattern could be fixed when the confinement is released, which is challenging, but remains significant for potential applications in microfabrication. To check this point, more experiments were carried out. Different from the above tests, in this case, the experiment was carried out on a cold platform. As shown in Supplementary Fig. 3a, a square liquid metal (Galinstan) droplet was first obtained. Then, the temperature of the substrate was decreased to – 20°C and kept for 20 min. During this period, solidification happens. After that, the upper glass plate was completely removed, and the square shape of the film was kept, as shown in Supplementary Fig. 3b. Moreover, when we used a needle to touch the square film, scratches appeared which suggests that the square film was fixed as a solid piece. In other words, the solidification of the polygonal liquid metal suggests our method would find applications in microfabrication. However, when making a comparison between Supplementary Figs. 3a and 3b, it is noted that the experimental process slightly affects the shape and size of the liquid film. The deviation between the solidification and the original liquid film is still an interesting topic for further scrutiny.

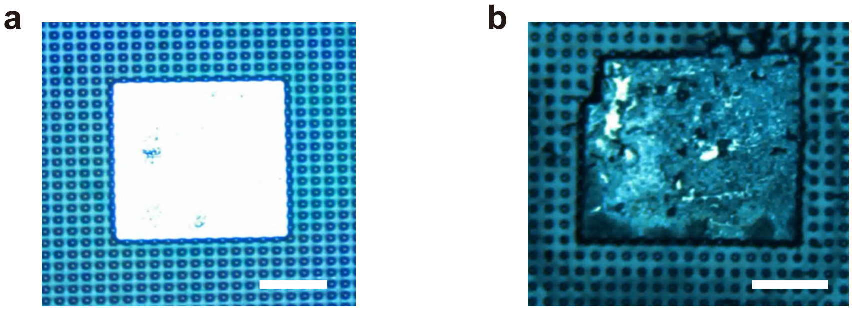

Supplementary Figure 3. Images of the patterns of the liquid metal (Galinstan). (**a**) Square pattern of a liquid metal droplet on a square arrangement of microtextures with a circular cross-section. (**b**) The shape of the liquid metal droplet was fixed after the confinement was removed. Scale bars are 500 μm. The diameter and the spacing of the pillars are 48.6 μm and 51.4 μm, respectively (Sample No. 12b in Supplementary Table 1).

Supplementary Discussion 2. Result of mercury droplet

It is worth discussing the generality of our method to create polygonal patterns using other liquid metals. As shown in Supplementary Fig. 4, when we replaced the Galinstan droplet by a mercury droplet, a square liquid film was well formed.

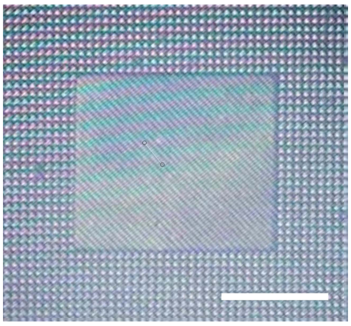

Supplementary Figure 4. Square pattern of a mercury droplet created by the square arrangement of microtextures with a circular cross-section. The scale bar is 500 μm. The diameter and the spacing of the pillars are 50 μm and 50 μm, respectively (Sample No. 12b in Supplementary Table 1).

Supplementary Discussion 3. Reversibility of the wetting state transition due to confinement

The reversibility of the wetting state transition of the polygonal droplets is checked in this section. Two examples of water and liquid metal droplets are presented in Supplementary Fig. 5a and b, respectively. The experimental processes are illustrated as follows. Before imposing confinement, the droplets were spherical (Supplementary Fig. 5(a-i) and (b-i)). Then, the upper superhydrophobic glass plate was lowered to force the spherical droplet to form polygonal patterns (Supplementary Fig. 5(a-ii) and (b-ii)). After that, the confinement was smoothly removed, the droplet recovered a spherical shape, suggesting a robust reversibility of the wetting state transition. During these processes, the droplets always keep the Cassie-Baxter wetting state. Moreover, the reversibility of the wetting state is available for all the experiments presented in the paper.

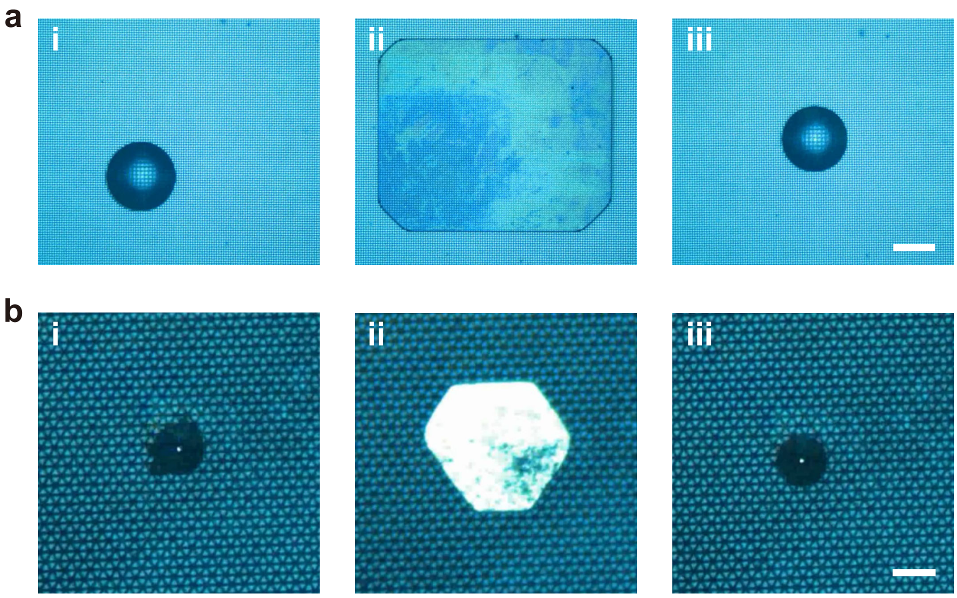

Supplementary Figure 5. Reversibility of the wetting state transition of water (**a**) and liquid metal (**b**) droplets. (**a-i**) After a water droplet was deposited on the substrate but before the confinement was imposed, the droplet demonstrated a spherical shape. (**a-ii**) A square liquid film was formed when the confinement was imposed. (**a-iii**) After the confinement was removed, the droplet recovered to a spherical shape. Scale bar, 500 μm. The sample is No. 12a in Supplementary Table 1. (**b-i**), (**b-ii**), (**b-iii**) Same procedures applied for a liquid metal (Galinstan) droplet. Scale bar, 100 μm. The sample is No. 1 in Supplementary Table 1.

Supplementary Discussion 4. Reproducibility of the polygonal droplet pattern

The reproducibility of the wetting state is demonstrated as follows. As shown in Supplementary Fig. 6, the morphologies of a water droplet are given. Specifically, the upper superhydrophobic glass plate was lowered to a certain height H ($H > H_c$) and then lifted, and the processes were repeated three times. Supplementary Fig. 6a, b and c show the corresponding moments of the water pattern under the certain value of H. Generally, the appearances of the pattern are very similar to each other, which suggests a good reversibility of the wetting state.

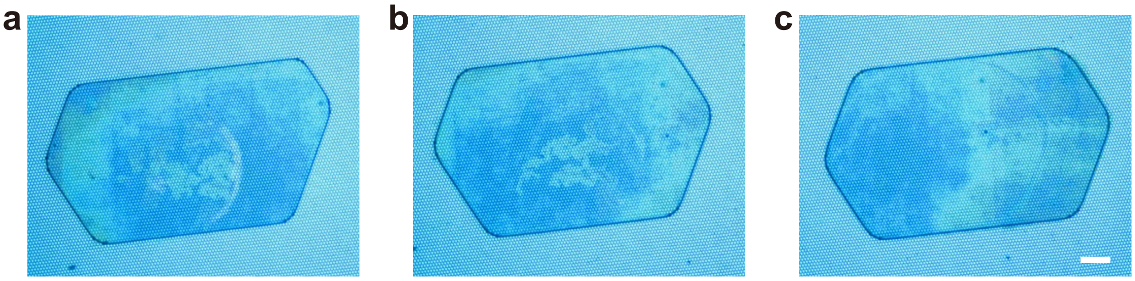

Supplementary Figure 6. Snapshots of the hexagonal shape of a water droplet. The glass plate repeatedly imposes and releases pressure on the droplet. (**a**), (**b**) and (**c**) show the configurations of the hexagonal shape, corresponding to the first, second and third times that the minimum separation H between the substrate and the glass reaches the same value. Scale bars are 250 μm. The sample is No. 6 in Supplementary Table 1.

Moreover, the reproducibility of the wetting state is also checked for the liquid metal (Galinstan), and the results are demonstrated in Supplementary Fig. 7. The superhydrophobic glass plate was repeatedly lowered and lifted three times. Supplementary Fig. 7a, b and c show the corresponding appearances of the liquid patterns with the same value of the separation H ($H > H_c$) between the glass plate and the substrate. The patterns are similar to each other. However, the reproducibility of the wetting state of the liquid metal is not as good as water. One possible reason is that the separation between the glass plate and the substrate in each confinement did not exactly have the same value. The liquid metal (Galinstan) has a much higher surfaces tension (624 mN/m) than water (72 mN/m), in order to create polygonal patterns, the liquid metal droplets have to be much highly confined compared with water droplets. Optimization of the experimental setup will a much higher precision will be the objective of our future work.

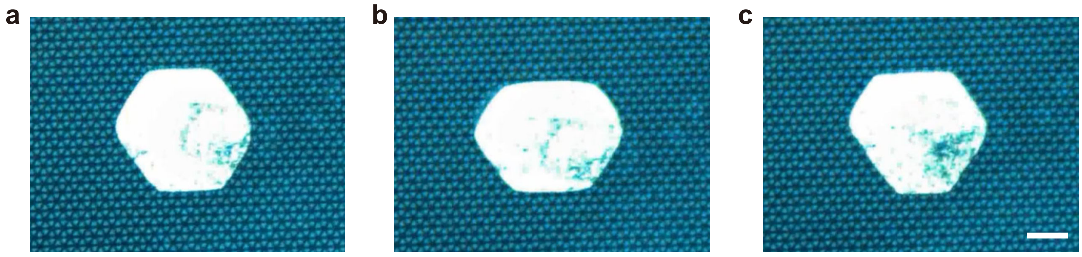

Supplementary Figure 7. Snapshots of the hexagonal shape of a liquid metal droplet. The glass plate repeatedly imposes and releases pressure on the droplet. (**a**), (**b**) and (**c**) show the configurations of the hexagonal liquid film pattern, corresponding to the first, second and third times that the minimum separation H between the substrate and the glass reaches the same value. Scale bars are 100 μm. The sample is No. 1 in Supplementary Table 1.

Supplementary Discussion 5. Diagram for more types of micropillared structures

In Fig. 2 of the main paper, for square pillars arranged in hexagonal arrays, diagrams to illustrate the liquid-vapor line fraction $\varepsilon(\alpha) = L_{LV}/L$ as the function of α are given. As shown in Supplementary Fig. 8, the diagrams for various cases are given. For the sake of simplicity, only one reference point (i.e., red dot in the left image of each panel) was chosen. Considering each polygon is symmetric, one half of each of them was considered. Supplementary Fig. 8a gives the appearance of triangular pillars arranged in square arrays. Based on $\varepsilon(\alpha)$, the block directions are known to appear in four directions, i.e., $\alpha \approx 0°, \pm 90°$ and $180°$. The appearance of the droplet pattern in our experiments confirms the theory. Supplementary Fig. 8b and Fig. 8c also suggest the agreement between the theoretical and experimental results. However, in Supplementary Fig. 8c, the droplet pattern is closer to a triangle, rather than a hexagon. Based on the discussion in the main paper (similar to Fig. 4), it is known that the sharpness of the corner of the polygonal droplet pattern is determined by the spacing b of the pillars. The spacing b in Supplementary Fig. 8c (b = 11 μm, No. 3 in Supplementary Table 1) is quite large, so apparently the corner of the droplet is not very obvious. Further discussions will be carried out in our future work.

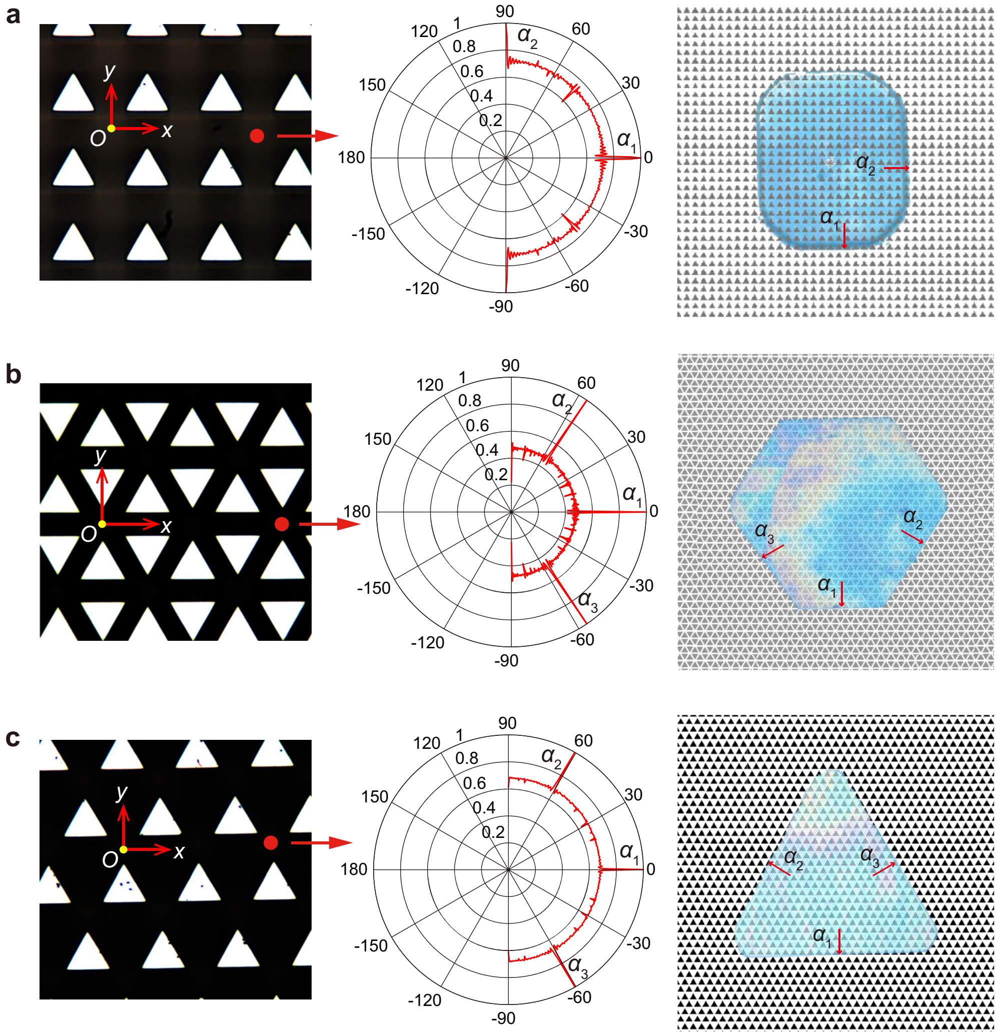

Supplementary Figure 8. Diagram illustrating the liquid-vapor line fraction $\varepsilon(\alpha) = L_{LV}/L$ as functions of α for triangular pillars arranged in various arrays. Polar coordinate systems are employed. (**a**) Top view image of the structure, diagram and droplet pattern for sample No. 2 in Supplementary Table 1. Four local maximum values of $\varepsilon_{max}(\alpha)$ corresponding to α = 0°, 180° and ± 90° are observed. (**b**) is for sample No. 1 in Supplementary Table 1 with six local maximum values of $\varepsilon_{max}(\alpha)$, corresponding to α = 0°, 180°, ± 56° and ± 124°. (**c**) is for sample No. 3 in Supplementary Table 1 with six local maximum values of $\varepsilon_{max}(\alpha)$, corresponding to α = 0°, 180°, ± 60° and ± 120°. The coordinate systems are given. Source data are provided as a Source Data file.

Supplementary Discussion 6. Theoretical analysis of the geometry of the droplet

To explain the result which had been obtained in Fig. 5 of the main paper. As shown in Supplementary Fig. 9, the contour of the hexagonal drop from the top view is given. The contour consists of six straight lines (marked as E), as well as a sixth-circle (marked as F) on each corner, and there is a simple relation $F = \pi R_{m,2}/3$. The area A enclosed by the contour could be categorized into three parts: (i) a hexagon (magenta color) with side length E and area A_1; (ii) six rectangles (green color as a representative one) with side length E and $R_{m,2}$, and area A_2 for each of them; (iii) six one-sixth sectors with radius $R_{m,2}$ (orange color as a representative one) and area A_3 for each of them.

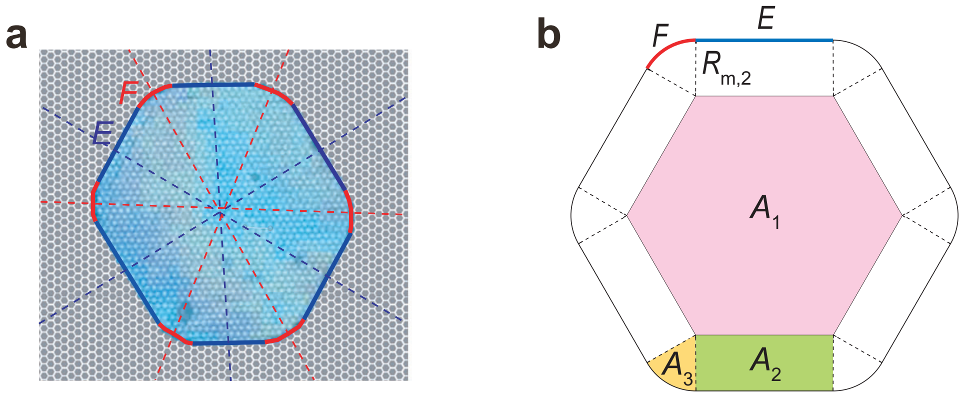

Supplementary Figure 9. Hexagonal liquid with sides and corners. (**a**) Experimental result from the top view. (**b**) Diagram and modelling illustrating that the liquid profile consists of three parts: a hexagon (magenta color) with area A_1, six rectangles with area A_2 for each (green color), and six one-sixth sectors with area A_3 for each (orange color).

The liquid volume conservation leads to the following relationship

$$A = \frac{V_0}{H} = A_1 + 6A_2 + 6A_3 = \frac{3^{3/2}}{2}E^2 + 6ER_{m,2} + \pi R_{m,2}^2, \quad \text{(S1)}$$

where V_0 is the volume of the droplet. Thus, the length of E is obtained as

$$E = \frac{2R_{m,2}}{\sqrt{3}}\left[\sqrt{1 + \frac{\sqrt{3}}{6}\left(\frac{V_0}{HR_{m,2}^2} - \pi\right)} - 1\right]. \quad \text{(S2)}$$

Since $F = \pi R_{m,2}/3$, by employing Eq. (3) in the main text, the ratio F/E as the function of V_0, H and θ_A can be obtained

$$\frac{F}{E}=\frac{\sqrt{3}\pi}{6}\frac{1}{\left[\sqrt{1+\frac{\sqrt{3}}{6}\left(\frac{V_0}{H^3}\left(1+\cos\theta_A\right)^2-\pi\right)}-1\right]}, \approx \frac{3}{\sqrt{1+\frac{3V_0}{H^3}\left(1+\cos\theta_A\right)^2}-3.28} \quad (S3)$$

where θ_A is the advancing contact angle. Strictly speaking, $A = V_0/H$ is only valid when H/H_0 << 1, so an empirical correction with a single fitting prefactor has to be employed, and Supplementary Eq. (S3) is rewritten into

$$\frac{F}{E}\approx\frac{3}{\sqrt{1+\frac{3V_0}{H^3}\left(1+\cos\theta_A\right)^2}-1}. \quad (4)$$

As shown in Fig. 5a of the main paper, the result of Eq. (4) (red curve) agree very well with the experimental data (black and red dots) with no adjustable parameter.

As shown in Fig. 5a of the main paper, we have given the relation between F/E and H/H_0. In this case, the diameter of the cross-sectional area, the spacing and the height of the cylindrical pillars are 17.4 μm, 7.6 μm and 90 μm, respectively (Sample No. 13 in Supplementary Table 1). Considering $f = 0.44$ and $\theta_{0,A} = 120°$, we obtain $\theta_A = 141°$ through Eq. (1) in the main paper.

Furthermore, Eq. (4) leads to two scaling regimes.

(1) When the thickness of the droplet is very small, i.e., H/H_0 << 1, Eq. (4) degrades into

$$\frac{F}{E} \approx \left. \frac{3}{\sqrt{1+\frac{3V_0}{H^3}\left(1+\cos\theta_{\mathrm{A}}\right)^2}-1} \right|_{H/H_0<<1}, \tag{S4}$$
$$\approx \frac{1.73}{\left(1+\cos\theta_{\mathrm{A}}\right)} \frac{H_0^{3/2}}{V_0^{1/2}} \cdot \left(\frac{H}{H_0}\right)^{3/2}$$

which leads to a $F/E \sim (H/H_0)^{3/2}$ scaling relation.

(2) When $H < H_0$ but the value of H/H_0 is not so small, since the term $(1 + \cos\theta_{\mathrm{A}})^2$ is very small, by employing Taylor series, the following relationship is obtained

$$\frac{F}{E} \approx \frac{3}{\sqrt{1+\frac{3V_0}{H^3}\left(1+\cos\theta_{\mathrm{A}}\right)^2}-1} \approx \frac{2}{\left(1+\cos\theta_{\mathrm{A}}\right)^2} \frac{H_0^3}{V_0} \cdot \left(\frac{H}{H_0}\right)^3, \tag{S5}$$

which leads to a $F/E \sim (H/H_0)^3$ scaling relation.

However, the experimental data do not cover a wide enough range of values of H/H_0 based on the present setup, more systematical experiments for smaller and larger values of H/H_0 need to be carried out in the future. For the other droplet patterns such as the triangular and square shapes, similar calculations can be carried out based on the above theoretical framework.

Supplementary Discussion 7. Spreading priority of the liquid

In addition to Fig. 6 in the main paper about the spreading priority of the contact line, as shown in Supplementary Fig. 10, more cases of the evolution of the droplet pattern are given. In Supplementary Fig. 10a, because the pillars arrange in the hexagonal pattern, when the droplet pattern is forming, the contact lines move synchronously (Supplementary Movie 5). However, as shown in Supplementary Fig. 10b, for the surface consisting of triangular pillars but arranged in the square pattern, when the square droplet pattern is forming, the four sides of the droplet pattern do not spread synchronously (Supplementary Movie 10). It seems that the upper and lower sides (which are parallel to the transverse sides of single pillars) of the droplet pattern pin, but the left and right sides of the droplet spread transversely.

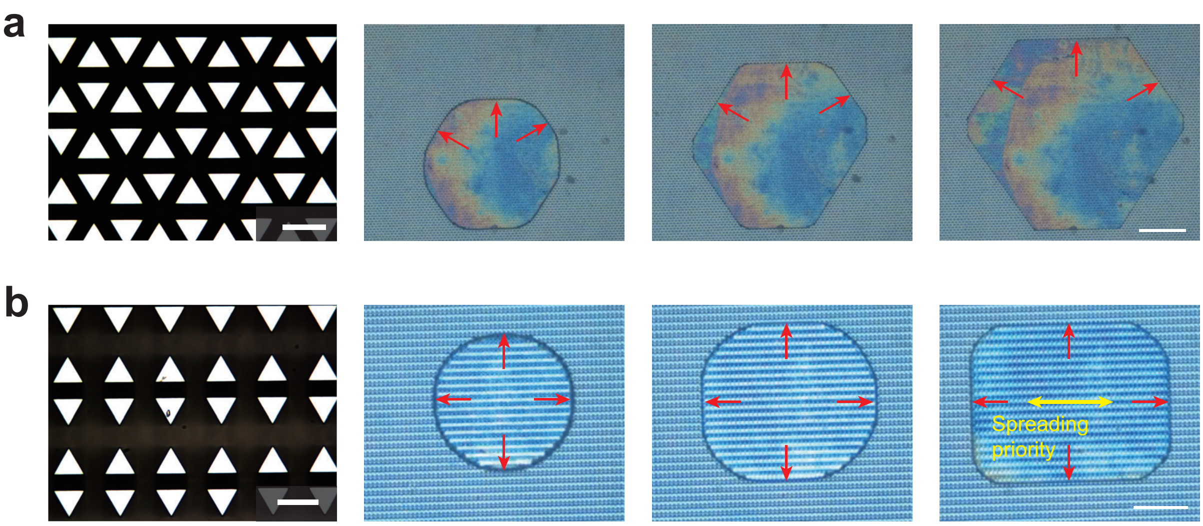

Supplementary Figure 10. Evolution of the morphology and spreading priority of the contact line on surfaces with a triangular cross-section of the pillars and various arrangements of the arrays. (**a**) Hexagonal droplet pattern created by the hexagonal arrangement of microtextures. (**b**) Square droplet pattern created by the square arrangement of microtextures. The red arrows represent the blocking directions. The yellow arrows in (**b**) represent the directions of spreading priority. Scale bar of the pillars 20 μm. Scale bar of the liquid patterns, 250 μm.

Supplementary Movie. Description of the Supplementary movie

All the movies were captured from the top view. The frame rate of the CCD camera is 30 frames per second (fps). When a droplet with a certain volume V_0 was deposited on the substrate, it adopts a circular solid-liquid-vapor three-phase contact line and a certain height H_0. After that, the superhydrophobic glass plate was smoothly declined to compress the droplet, and a polygonal droplet pattern appears. At the moment that the separation between the substrate and the glass plate reached a critical value H_c, the collapse happened. Detailed information of the movies is listed in Supplementary Table 2.

Supplementary Table 2. Detailed information of the Supplementary movies. "Sample No." followed "Movie No." represents the movie corresponding to the number of the sample in Supplementary Table 1. The corresponding figures in the main paper and the Supplementary Information (SI) are given. The unit of the droplet volume is nl. The scale bar is 500 μm in each movie.

Movie No.	Sample No.	Main paper	SM	V_0 (nl)	H_0 (μm)	H_c (μm)
1	3	Fig. 1b(i), Fig. 4a, Fig. 6b	SI Fig. 2a(3), SI Fig. 8a	28	366	43.2
2	12a	Fig 1b(ii), Fig. 4b	SI Fig. 2d(12)	26.5	363	24.3
3	5	Fig. 1b(iii), Fig. 4c	SI Fig. 2b(5)	32.9	350	11.9
4	2	Fig. 1c, Fig. 6a	SI Fig. 2a(2), SI Fig. 8c	53.2	434	31
5	1	Fig. 1d(i)	SI Fig. 2a(1), SI Fig. 8b, SI Fig. 10a	12.8	242	11.9
6	6	Fig. 1d(ii)	SI Fig. 2b(6)	19.5	283	13.9
7	9	Fig. 1d(iii)	SI Fig. 1b, SI Fig. 2c(9)	25.8	326	12.3
8	13	Fig. 1d(iv), Fig. 3d, Fig. 5	SI Fig. 2d(13), SI Fig. 9	24	318	13.6
9	7	Fig. 2	SI Fig. 2b(7)	43	381	28.2
10	4	- -	SI Fig. 2a(4), SI Fig. 10b	32.3	378	34.3
