# Supplementary material for: Polygonal non-wetting droplets on microtextured surfaces
Source: Nat Commun. 2022 May 13;13:2685. doi: 10.1038/s41467-022-30399-0 (PMC9106735; doi:10.1038/s41467-022-30399-0)
Supplement: Supplementary file 2 — Description of Additional Supplementary Files [file 41467_2022_30399_MOESM2_ESM.pdf]

Title: Supplementary Movie 1:
Description: Evolution of the liquid film on Sample No. 3 in Supplementary Table 1. The polygonal pattern is corresponding to Figures 1b(i), 4a, 6b in the main manuscript and Supplementary Figures 2a(3), 8a. The volume of the droplet is 28 nl. The frame rate of the CCD camera is 30 frames per second (fps).

Title: Supplementary Movie 2:
Description: Evolution of the liquid film on Sample No. 12a in Supplementary Table 1. The polygonal pattern is corresponding to Figure 1b(ii), 4b in the main manuscript and Supplementary Figures 2d(12). The volume of the droplet is 26.5 nl. The frame rate of the CCD camera is 30 frames per second (fps).

Title: Supplementary Movie 3:
Description: Evolution of the liquid film on Sample No. 5 in Supplementary Table 1. The polygonal pattern is corresponding to Figure 1b(iii), 4c in the main manuscript and Supplementary Figures 2b(5). The volume of the droplet is 32.9 nl. The frame rate of the CCD camera is 30 frames per second (fps).

Title: Supplementary Movie 4:
Description: Evolution of the liquid film on Sample No. 2 in Supplementary Table 1. The polygonal pattern is corresponding to Figure 1c, 6a in the main manuscript and Supplementary Figures 2a(2), 8c. The volume of the droplet is 53.2 nl. The frame rate of the CCD camera is 30 frames per second (fps).

Title: Supplementary Movie 5:
Description: Evolution of the liquid film on Sample No. 1 in Supplementary Table 1. The polygonal pattern is corresponding to Figure 1d(i) in the main manuscript and Supplementary Figures 2a(1), 8b, 10a. The volume of the droplet is 12.8 nl. The frame rate of the CCD camera is 30 frames per second (fps).

Title: Supplementary Movie 6:
Description: Evolution of the liquid film on Sample No. 6 in Supplementary Table 1. The polygonal pattern is corresponding to Figure 1d(ii) in the main manuscript and Supplementary Figure 2b(6). The volume of the droplet is 19.5 nl. The frame rate of the CCD camera is 30 frames per second (fps).

Title: Supplementary Movie 7:
Description: Evolution of the liquid film on Sample No. 9 in Supplementary Table 1. The polygonal pattern is corresponding to Figure 1d(iii) in the main manuscript and Supplementary Figures 1b, 2c(9). The volume of the droplet is 25.8 nl. The frame rate of the CCD camera is 30 frames per second (fps).

Title: Supplementary Movie 8:
Description: Evolution of the liquid film on Sample No. 13 in Supplementary Table 1.

The polygonal pattern is corresponding to Figures 1d(iv), 3d, 5 in the main manuscript and Supplementary Figures 2d(13), 9. The volume of the droplet is 24 nl. The frame rate of the CCD camera is 30 frames per second (fps).

Title: Supplementary Movie 9:
Description: Evolution of the liquid film on Sample No. 7 in Supplementary Table 1. The polygonal pattern is corresponding to Figure 2 in the main manuscript and Supplementary Figures 2b(7). The volume of the droplet is 43 nl. The frame rate of the CCD camera is 30 frames per second (fps).

Title: Supplementary Movie 10:
Description: Evolution of the liquid film on Sample No. 4 in Supplementary Table 1. The polygonal pattern is corresponding to Supplementary Figures 2a(4), 10b. The volume of the droplet is 32.3 nl. The frame rate of the CCD camera is 30 frames per second (fps).
